# The Kaiser score reliably excludes malignancy in benign contrast-enhancing lesions classified as BI-RADS 4 on breast MRI high-risk screening exams

**DOI:** 10.1007/s00330-020-06945-z

**Published:** 2020-06-06

**Authors:** Ruxandra Iulia Milos, Francesca Pipan, Anastasia Kalovidouri, Paola Clauser, Panagiotis Kapetas, Maria Bernathova, Thomas H. Helbich, Pascal A. T. Baltzer

**Affiliations:** 1 Department of Biomedical Imaging and Image-guided Therapy, Division of Molecular and Gender Imaging, Medical University of Vienna, Waehringer-Guertel 18-20, A-1090 Vienna, Austria; 2 Institute of Diagnostic Radiology, University of Udine, Udine, Italy; 3 Radiology Department, Geneva University Hospital, Geneva, Switzerland

**Keywords:** Breast cancer, Magnetic resonance imaging, Sensitivity and specificity, Decision support systems, Clinical, Screening

## Abstract

**Objectives:**

MRI is an integral part of breast cancer screening in high-risk patients. We investigated whether the application of the Kaiser score, a clinical decision-support tool, may be used to exclude malignancy in contrast-enhancing lesions classified as BI-RADS 4 on breast MRI screening exams.

**Methods:**

This retrospective study included 183 consecutive, histologically proven, suspicious (MR BI-RADS 4) lesions detected within our local high-risk screening program. All lesions were evaluated according to the Kaiser score for breast MRI by three readers blinded to the final histopathological diagnosis. The Kaiser score ranges from 1 (lowest, cancer very unlikely) to 11 (highest, cancer very likely) and reflects increasing probabilities of malignancy, with scores greater than 4 requiring biopsy. Receiver operating characteristic (ROC) curve analysis was used to evaluate diagnostic accuracy.

**Results:**

There were 142 benign and 41 malignant lesions, diagnosed in 159 patients (mean age, 43.6 years). Median Kaiser scores ranged between 2 and 5 in benign and 7 and 8 in malignant lesions. For all lesions, the Kaiser score’s accuracy, represented by the area under the curve (AUC), ranged between 86.5 and 90.2. The sensitivity of the Kaiser score was high, between 95.1 and 97.6% for all lesions, and was best in mass lesions. Application of the Kaiser score threshold for malignancy (≤ 4) could have potentially avoided 64 (45.1%) to 103 (72.5%) unnecessary biopsies in 142 benign lesions previously classified as BI-RADS 4.

**Conclusions:**

The use of Kaiser score in high-risk MRI screening reliably excludes malignancy in more than 45% of contrast-enhancing lesions classified as BI-RADS 4.

**Key Points:**

• *The Kaiser score shows high diagnostic accuracy in identifying malignancy in contrast-enhancing lesions in patients undergoing high-risk screening for breast cancer.*

• *The application of the Kaiser score may avoid > 45% of unnecessary breast biopsies in high-risk patients.*

• *The Kaiser score aids decision-making in high-risk breast cancer MRI screening programs.*

**Electronic supplementary material:**

The online version of this article (10.1007/s00330-020-06945-z) contains supplementary material, which is available to authorized users.

## Introduction

MRI provides the highest sensitivity for the detection of breast cancer [1–5] and it plays a central role in the screening of patients with a hereditary or familial high-risk for developing breast cancer [6]. To achieve a significant risk reduction, either prophylactic bilateral mastectomy or annual screening is provided in the high-risk population [7, 8]. Moreover, women at an increased risk for the development of breast cancer are usually prone to develop breast cancer at a much younger age [7] and are consequently screened from a younger age and for a longer period of time. Although these patients usually undergo multimodality screening, it has been shown that MRI is the best modality with which to detect familial breast cancer, regardless of patient age, breast density, or risk status [9, 10]. An important proportion of these lesions are MRI-only lesions [9] and it has been shown that MRI particularly detects the small (less than 10 mm in diameter) and more aggressive types of breast cancer [11]. However, it has been postulated that the imaging characteristics of cancer that develops in women at very high-risk are less specific and may resemble benign lesions (fibroadenoma-like masses and benign kinetic features) [12, 13]. Consequently, on the basis of these results, it has been recommended that, in high-risk women, small enhancing lesions should be regarded with suspicion and biopsied, or patients should be followed up at 6 months [13]. The BI-RADS lexicon can be used to describe enhancing breast lesions in a standardized and commonly understandable way.

While the BI-RADS lexicon provides a common language for lesion description in a standardized and structured approach [14, 15], it does not provide guidance on how lesions that present with certain features should be managed. The Kaiser score is able to fill this gap [16, 17]; it is a clinical decision rule that combines BI-RADS features in a simple machine-learning derived flowchart. Following the flowchart results in a diagnostic score that reflects the increasing probabilities of malignancy, ranging from 1 to 11, with scores greater than 4 requiring biopsy. As the Kaiser score combines several criteria to achieve a diagnosis, we hypothesized that the cancers detected in high-risk women could objectively be diagnosed as such using the Kaiser score, even though they might present with a circumscribed appearance that was referred to as “fibroadenoma-like” in prior works.

Consequently, we assessed the ability of the Kaiser score to diagnose malignancy in a consecutive population of histologically proven suspicious (MR BI-RADS 4), contrast-enhancing lesions diagnosed in a high-risk breast cancer patient screening program.

## Methods

### Study population

This study is a retrospective single-center investigation of a prospectively populated high-risk screening database. All participants in the study prospectively provided written, informed consent to the examination and use of their data and the study was approved by the local institutional review board (Medical University of Vienna). The need for additional informed consent of this retrospective analysis of the imaging data was waived by the IRB. The study included high-risk women with a proven mutation in one of the breast cancer susceptibility genes (BRCA-1 or BRCA2) or those who fulfilled the criteria of increased familial risk as described previously [9, 14, 18, 19]. The family history inclusion criteria for high-risk screening in Austria are the following: (a) three breast cancers at age ≤ 60 years; (b) two breast cancers at age ≤ 50 years; (c) one breast cancer at age ≤ 35 years; (d) one breast cancer at age ≤ 50 years and one ovarian cancer at any age; (e) two ovarian cancers at any age; and (f) one male and one female cancer at any age. All the affected first-degree relatives must be on the same side of the family. A woman’s personal cancer history can contribute to the criteria. Women who fulfilled these family history criteria were advised to undergo genetic testing at our institution, but remained within the study even if they decided not to be tested or if the tests were negative for a breast cancer susceptibility gene.

All study patients underwent annual screening, consisting of two-view mammography, ultrasound, and MR imaging of the breast every 12 months, with a maximum interval of 1 month between the individual modalities [9, 19].

From our prospectively populated, high-risk screening database, we selected all 197 consecutive patients from February 2003 to August 2015 (mean age, 43.6 ± 11.2 years; age range, 23–80) who underwent 257 image-guided biopsies (either ultrasound-, stereotactic-, or MRI), both core needle biopsy (CNB) and vacuum-assisted breast biopsy (VABB), at our institution, a tertiary care university hospital. Excluded were all cases that underwent biopsy due to findings not visible on MRI (e.g., mammographic or sonographic abnormalities that did not present as enhancing lesions on MRI), all patients with lesions in which localization was not clear upon retrospective review (e.g., patients who underwent ultrasound-guided biopsy of subtle anomalies which could not be connected to a localized enhancing lesion on MRI), and patients whose diagnostic MR images could not be retrieved electronically, as they were either corrupted or stored on non-compatible DICOM storage. Details are given in the patient and lesion selection flowchart (see supplementary Figure 1). The final study database consisted of 183 breast MRI-visible lesions classified as BI-RADS 4 in 159 patients (mean age, 43.6 ± 11.6 years; age range, 24–80). Parts of our institutional high-risk screening database were used in prior publications [9, 14], with substantially different rationales and results.

### Imaging and image-guided biopsies

Until September 2008, MRI of the breast was performed on a 1.0-T scanner with a dedicated double breast coil (Gyroscan T10-NT; Philips). The MRI sequence protocol consisted of a sagittal T2-weighted STIR sequence and axial, T1-weighted, three-dimensional, gradient-echo dynamic sequences. Images were obtained once before intravenous contrast agent administration and six times at intervals of 70 s thereafter. After September 2008, a 1.5-T MRI scanner MAGNETOM Avanto (Siemens) was used. After a sagittal T2-weighted sequence with fat suppression (turbo inversion recovery magnitude), axial T1-weighted dynamic sequences were measured once before and four times after contrast agent injection at intervals of 90 s. In 2013, the protocol was modernized, changing the axial dynamics to a high-spatial-resolution, Dixon fat-suppressed VIBE sequence while maintaining the temporal resolution of 90 s. In addition, precontrast axial T2w-TSE, STIR, and DWI sequences were introduced as recommended in [16].

To minimize hormone-related background breast tissue enhancement, premenopausal women were scheduled on the seventh to the fourteenth day of their menstrual cycle [8].

All lesions classified as BI-RADS 4 (suspicious) were biopsied using image guidance (either CNB or VABB was performed according to already established guidelines [20–23]) or surgically biopsied [24]. All biopsy specimens underwent histopathological analysis, the gold standard of our study. Histopathological tissue analysis was performed by an experienced, board-certified breast pathologist (M.R.). The B classification for diagnosis was applied [25]. In all patients with malignant lesions, i.e., invasive carcinoma and/or ductal carcinoma in situ (DCIS), and in lesions with uncertain malignant potential (histopathological B3), surgical biopsy was performed, after wire localization. In case of a benign finding at histopathology, the patients underwent follow-up with breast MRI at 12 months (according to the annual high-risk screening).

### Data analysis

All 183 included cases were independently analyzed by three breast imaging radiologists, who were blinded to the final histopathological diagnosis. The readers were breast fellowship–trained radiologists trained at three different institutions. All had similar prior experience between 3 and 5 years.

Masses were classified according to their BI-RADS lexicon appearance into mass, non-mass, and foci. The readers were asked to classify all biopsied lesions using the Kaiser score as described in the literature [16] and did not undergo formal training before reading the study cases. This score combines five independent morphological and kinetic BI-RADS lexicon-derived descriptors (internal enhancement, lesion margins, presence of spiculations (formerly referred to as “root sign” [14]), SI-time-curve type, and presence of edema) in a flowchart-like algorithm. The resulting score reflects the increasing probabilities of malignancy (1 = lowest, cancer very unlikely to 11 = highest, cancer very likely) [16]. Scores greater than 4 require biopsy. A diagnostic category was assigned for each biopsied lesion.

### Statistical analysis

Statistical analysis was performed by P.B. using SPSS 23.0 (SPSS, IBM) and MedCalc 18 (MedCalc software bvba). A receiver operating characteristic (ROC) analysis was performed and the area under the ROC curve was measured to determine overall diagnostic performance. Sensitivity and specificity were calculated at a cutoff value of > 4, which indicated malignancy. Inter-reader agreement of the dichotomized (Kaiser scores 1–4 were considered benign, 5–11 malignant) Kaiser score readings was assessed using kappa statistics. Cross-tabulated data were compared by the chi-squared test. *p* values ≤ 0.05 were considered statistically significant.

## Results

### Lesion characteristics

The study cohort included 159 patients with 183 histologically verified lesions (see supplementary Figure 1). In total, 121 of these lesions were examined and biopsied before 2009, 62 afterwards. The mean size of the 41 malignant lesions (17.5 ± 13.8 mm) was significantly higher than that of the 142 benign lesions (11–6 ± 7.5 mm, *p* = 0.010, Mann-Whitney *U* test). There were 88 mass lesions, 48 non-mass lesions, and 47 foci. Of all 88 mass lesions, 24 (27.3%) were malignant and 64 benign. Of the 48 non-mass lesions, 10 (20.8%) were malignant and 38 benign. Seven (14.9%) of the 47 foci were malignant and 40 benign. Detailed histopathological diagnoses and subtypes are given in Table 1.

**Table 1:** Final histological characteristics of the biopsied lesions

	Subtype	*n* (%)
Benign		142/183 (77.6%)
	Adenosis, sclerosing adenosis	41/142 (28.9%)
	Fibroadenoma, fibroadenomatoid hyperplasia	23/142 (16.2%)
	Benign epithelial proliferations	41/142 (28.9%)
	Benign breast tissue, pseudoangiomatous stromal hyperplasia	25/142 (17.6%)
	Inflammation	1/142 (0.7%)
	B3 lesion	11/142 (7.7%)
Malignant		41/183 (22.4%)
	**DCIS**	12/41 (29.3%)
	Luminal A type	7/12 (58.3%)
	Luminal B type	0/12 (0%)
	Her2 type	5/12 (41.7%)
	Triple negative	0/12 (0%)
	**Invasive cancer**	29/41 (70.7%)
	Luminal A type	11/29 (38.0%)
	Luminal B type	6/29 (20.7%)
	Her2 type	3/29 (10.3%)
	Triple negative	9/29 (31.0%)

### Inter-reader agreement

**Fig. 1:**
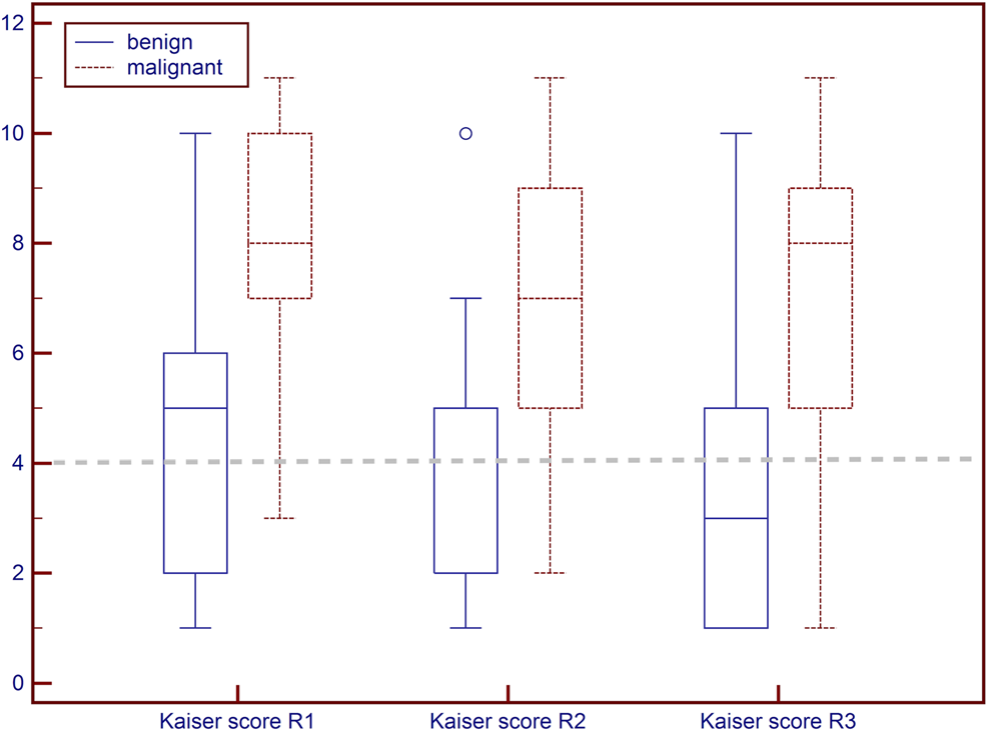
Boxplot of Kaiser score distribution for all three readers (R1, R2, R3) stratified by final diagnosis as benign or malignant. The grey dashed line indicates the biopsy recommendation threshold. It is evident that a majority of benign lesions presents with Kaiser scores below this threshold while most malignant lesions present with Kaiser scores above 4

The kappa agreement among the three readers for the characterization of breast lesions according to the Kaiser score was fair to moderate (R1 vs R2, 0.393; R1 vs R2, 0.362; R2 vs R3, 0.560). The median Kaiser scores in benign lesions were 5, interquartile range (IQR) 2–6 (R1); 2, IQR 2–5 (R2); and 3, IQR 1–5 (R3). In malignant lesions, median Kaiser scores were 8, IQR 7–10 (R1); 7, IQR 5–9 (R2); and 8, IQR 5–9 (R3) (see Fig. 1**)**.

### ROC curve analyses

**Fig. 2:**
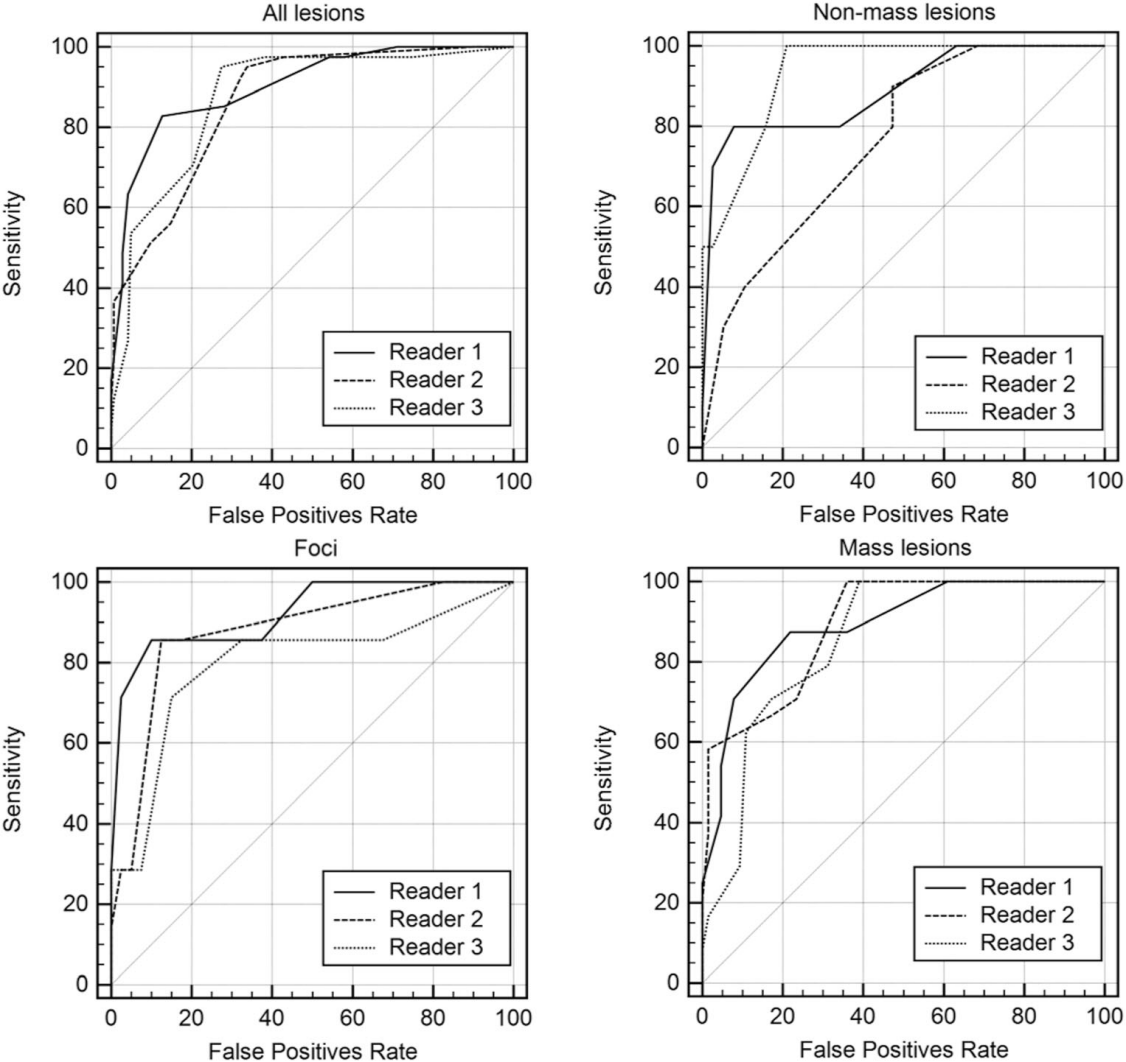
Receiver operating characteristic (ROC) curves for all three readers. All lesions are given in the upper left, non-mass lesions in the upper right, foci in the lower left, and mass lesions in the lower right. Diagnostic performance estimates are summarized in Table 2

Detailed results about the diagnostic performance of the Kaiser score in all lesions, masses, non-mass lesions, and foci are shown in Tables 2 and 3 and Fig. 2.

**Table 2:** Diagnostic performance indices for all three readers including subgroups of mass, non-mass lesions, and foci

	AUC	95% CI
All lesions (183)		
Reader 1	90.2	85.0–94.1
Reader 2	86.5	80.7–91.1
Reader 3	87.5	81.8–91.9
Mass (88)		
Reader 1	89.5	81.1–95.0
Reader 2	89.3	80.8–94.9
Reader 3	85.4	76.2–92.0
Non-mass (48)		
Reader 1	89.0	76.5–96.2
Reader 2	76.3	61.8–87.4
Reader 3	93.6	82.5–98.6
Foci (47)		
Reader 1	92.3	80.7–98.1
Reader 2	87.7	74.8–95.4
Reader 3	79.8	65.6–90.1

Values are given as percentages, absolute numbers in brackets; *AUC*, area under the receiver operating characteristics curve; *CI*, confidence interval

**Table 3:** Sensitivity and specificity for all three readers including subgroups of mass, non-mass lesions, and foci

	Sensitivity (TP/TP + FN)	95% CI	Specificity (TN/TN + FP)	95% CI
All lesions (183)				
Reader 1	97.6 (40/41)	87.1–99.9	45.1 (64/142)	37.4–54.3
Reader 2	92.7 (38/41)	83.5–99.4	67.6 (96/142)	59.2–75.2
Reader 3	95.1 (39/41)	80.1–98.5	72.5 (103/142)	64.4–79.7
Mass (88)				
Reader 1	100 (24/24)	85.8–100	39.1 (25/64)	27.1–52.1
Reader 2	100 (24/24)	85.8–100	64.1 (41/64)	51.1–75.7
Reader 3	100 (24/24)	85.8–100	60.9 (39/64)	47.9–72.9
Non-mass (48)				
Reader 1	100 (10/10)	69.2–100	36.8 (14/38)	21.8–54
Reader 2	80 (8/10)	44.4–97.5	52.6 (20/38)	35.8–69.0
Reader 3	100 (10/10)	69.2–100	79.0 (30/38)	62.7–90.4
Foci (47)				
Reader 1	85.7 (6/7)	42.1–99.6	62.5 (25/40)	45.8–77.3
Reader 2	85.7 (6/7)	42.1–99.6	87.5 (35/40)	73.2–95.8
Reader 3	71.4 (5/7)	29.0–96.3	85.0 (34/40)	50.9–81.4

Values are given as percentages, absolute numbers in brackets; *TP*, true positives; *TN*, true negatives; *FP*, false positives; *FN*, false negatives; *CI*, confidence interval

#### Area under the ROC-curve

Using the Kaiser score for all lesions, the overall accuracy, represented by the area under the curve (AUC), ranged between 86.5 and 90.2 (Table 2). Taken separately, for mass lesions, the AUC for lesion diagnosis ranged between 85.4 and 89.5, whereas in non-mass lesions, the AUC varied between 76.3 and 93.6. For foci, the AUC ranged between 79.8 and 92.3 (Table 2).

#### Sensitivity and false-negative findings

The sensitivity of the Kaiser score was high, between 92.7 and 97.6% for all lesions, 100% for mass lesions, and 80 to 100% for non-mass lesions, while for foci, it was lower, with 71.4 to 85.7% (Table 3). Four (two foci and two non-mass lesions) of the 41 malignant lesions were missed. One of the seven malignant foci (luminal A type invasive cancer) was missed by all three readers (Kaiser scores 3, 4, and 4, respectively). Reader 2 reported two additional false-negative non-mass lesions (one luminal A type invasive cancer, and one HER 2 type DCIS, Kaiser scores 3 and 4, respectively), while reader 3 failed to identify one additional focus as malignant (luminal A type invasive cancer, Kaiser score 1). All false-negative readings were diagnosed before 2009, when the scanner was changed from 1.0 to 1.5 T. Of the 41 cancers, 22 were diagnosed prior to 2009 and 19 afterward. The difference between false-negative findings before and after this date was not statistically significant (*p* > 0.05, respectively).

#### Specificity and the potential to avoid unnecessary biopsies

**Fig. 3:**
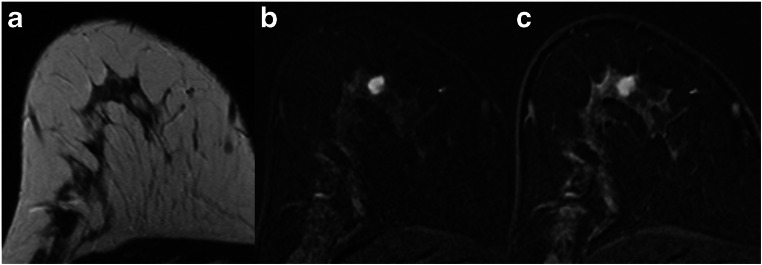
A 47-year-old high-risk patient: MRI (**a** T2w; **b**, **c** subtracted early and late contrast-enhanced, T1-weighted images) shows a rather circumscribed mass lesion with heterogeneous internal enhancement and wash-out, corresponding to a Kaiser score of 8. Histology revealed a luminal-type invasive lobular cancer, B5b

**Fig. 4:**
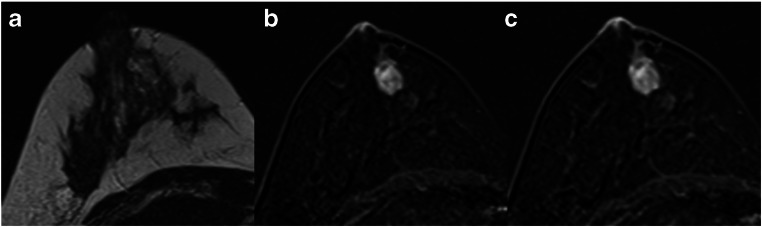
A 35-year-old high-risk patient: MRI (**a** T2w; **b**, **c** subtracted early and late contrast-enhanced, T1-weighted images) shows a circumscribed mass lesion with heterogeneous internal enhancement and wash-out, corresponding to a Kaiser score of 8. Note the hyperintense, fibroadenoma-like T2w-correlate (**a**). Histology revealed a triple-negative invasive ductal cancer, B5b

**Fig. 5:**
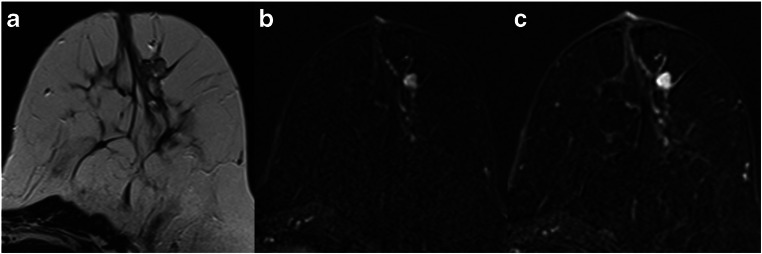
A 39-year-old high-risk patient: MRI (**a** T2w; **b**, **c** subtracted early and late contrast-enhanced, T1-weighted images) shows a circumscribed mass lesion with heterogeneous internal enhancement and persistent signal increase, corresponding to a Kaiser score of 1. Note the fibroadenoma-like T2w-correlate (**a**). Histopathology revealed a fibroadenoma, B2

**Fig. 6:**
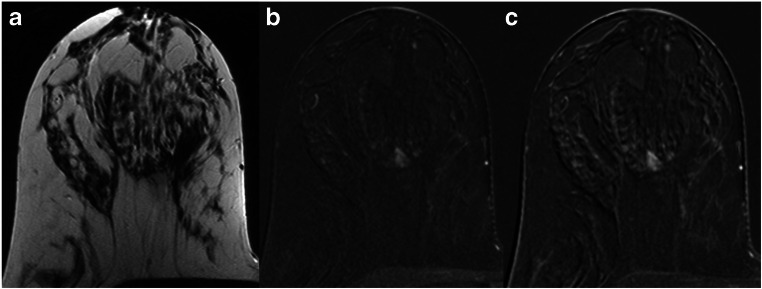
A 44-year-old high-risk patient: MRI (**a** T2w; **b**, **c** subtracted early and late contrast-enhanced, T1-weighted images) shows a non-circumscribed, non-mass lesion with heterogeneous internal enhancement and persistent signal increase, corresponding to a Kaiser score of 3. Histopathology revealed benign epithelial proliferations, B2

The specificity for all lesions ranged between 45.1 and 72.5% (Table 3). The application of the Kaiser score improved diagnosis by correctly identifying between 64 (45.1%) and 103 (72.5%) of 142 benign lesions previously classified as BI-RADS 4. Accordingly, 25 to 41 mass lesions (28.4 to 46.6%), 14 to 30 non-mass lesions (29.2 to 62.5%), and 25 to 34 foci (53.2 to 72.3%) could have been predicted using the Kaiser score with a cutoff value of 4. Thus, biopsies could have been avoided in a large percentage of cases. Examples are given in Figs. 3, 4, 5, and 6.

## Discussion

This study investigated the benefit of implementing the Kaiser score as a decision tool in MRI suspicious (BI-RADS 4) contrast-enhancing lesions diagnosed in patients at high-risk for developing breast cancer. This is clinically highly relevant as it refutes the notion of benign-appearing cancers in the investigated setting. Furthermore, we could show that the Kaiser score is applicable in high-risk patients independent of lesion appearance as mass, non-mass, or foci. The diagnostic performance equaled that of the Kaiser score applied in other scenarios [26–28]. The thresholds established in other indications could be reproduced, allowing exclusion of cancer with high certainty. Potentially, 45 to 72% of all unnecessary biopsies could have been avoided by applying the Kaiser score prior to biopsy.

The Kaiser score uses a small set of morphological and dynamically relevant features that were chosen by machine-learning methodology (presence of spiculations/root sign, enhancement kinetics, lesion margin, internal enhancement pattern, and ipsilateral edema). The result is a three-step flowchart with the score providing the probabilities of malignancy, ranging from 1 to 11. Thus, enhancing lesion assessment can be simplified and structured and the results can be used for evidence-based decision-making. Scores below 5 should be considered benign, while histological workup is mandatory for higher scores [16]. This was initially tested in an exploratory study on biopsy-proven lesions in a mixed study population [17] and thereafter validated in consecutive problem-solving cases [26], suspicious MRI-only lesions [27], and in lesions that presented as suspicious mammographic microcalcifications [28]. The application of the Kaiser score relies on generally recommended standard breast MRI protocols (T2-weighted sequences and dynamic, contrast-enhanced, T1-weighted sequences), and it was shown to be independent of the type of scanners/vendors used [27] and helpful for less experienced radiologists [26]. It does not require any additional functional imaging, such as DWI or MR spectroscopy, or postprocessing software [17]. Yet, it allows the integration of further diagnostic data, either clinical (such as bloody discharge), conventional findings (e.g., suspicious mammographic calcifications), or quantitative information (e.g., DWI), as discussed in [16].

We found that the Kaiser score is highly accurate in the setting of high-risk patients. All readers achieved a high sensitivity, with the only false-negative results in non-mass lesions and foci. This could be explained by the difficulty of determining the margin type or discerning the enhancement pattern in lesions smaller than 5 mm, especially on old examinations of a lower quality. Notably, although statistically not significant due to a low sample size, all false-negative ratings were obtained in examinations older than 10 years, stressing the importance of high image quality for interpretation of these lesions. The already established cutoff value for a biopsy recommendation in Kaiser scores exceeding 4 [16, 26, 27] was applicable in our study cohort. Thus, even if initially categorized as BI-RADS 4 lesions, scores of 4 or lower were robustly indicative of a benign outcome. Diagnostic tests are not perfect. If low Kaiser scores are applied to avoid unnecessary biopsies, this comes at the cost of false-negative findings: missed cancers. In healthcare, the application of a decision-making tool such as the Kaiser score is always an ethical issue: how many avoided unnecessary biopsies are worth one missed cancer? None of the false-negative lesions presented as masses on MRI. We think it is safe to conclude that the Kaiser score can without a doubt be safely applied to downgrade mass lesions but caution should be used when interpreting non-mass lesions and foci. The number of false-negative findings in this study was low: lesions were either luminal A type invasive cancer or DCIS. It can therefore be relatively safely assumed that downgrading a lesion would not have changed the patients’ prognosis but rather led to a delayed diagnosis in a biologically less significant malignancy. Patients in this setting undergo annual screening, equaling the maximum diagnostic delay. Whether such downgraded lesions should be primarily assigned BI-RADS 3 and undergo an additional follow-up at 6 months is discussed elsewhere [29].

The results once more corroborate the usefulness of a structured and evidence-based diagnostic approach. In high-risk MRI screening, the low prevalence of malignancy is connected to an inherent risk of false-negative findings [30]. Radiologists seemingly compensate for this by using a rather low biopsy threshold. Although the 5th BI-RADS lexicon edition [15] can be used for standardized lesion description [14], the results of our paper point out the limitations of empirical BI-RADS 4 category assignments that do not follow objective rules in high-risk patients.

Previous studies have shown that the imaging phenotypes of malignancy differed in women at high risk, with a high percentage of invasive cancers appearing as fibroadenoma-like masses, but without fibroadenoma-like internal enhancement or enhancement kinetics [12, 31]. However, our results demonstrate that there are no cancers with exclusively benign criteria. The structured combination of morphological and functional criteria provided by the Kaiser score avoids misinterpretations of a single diagnostic criterion such as circumscribed margins.

The combination of diagnostic criteria is available due to the multiparametric character of breast MRI. Recently, alternative, abbreviated protocols have been proposed for screening women with dense breast tissue [6, 32]. The aim is to reduce the scan time by acquiring only one pre-contrast and one early post-contrast T1-weighted image set. Consequently, the reader can obtain a quick overview of presence or absence of enhancement on a single, high-contrast, maximum intensity projection (MIP) image, followed by subsequent characterization of enhancement with respect to configuration, morphology, margins, and internal architecture based on an analysis of the individual subtracted images [32]. Nonetheless, the shape of the enhancement curve was shown to be relevant for estimating the probability of malignancy, increasing from a type I (persistent) to a type III (wash-out) curve. In the framework of the machine learning–derived Kaiser score, the enhancement curve type is the second most important diagnostic criterion. Thus, in the setting of a high-risk patient, with no information about the enhancement kinetics, a circumscribed lesion with enhancement must always be considered suspicious. Our study, therefore, provides indirect evidence against abbreviated, non-dynamic protocols for high-risk screening: due to the lack of diagnostic information provided by the enhancement kinetics, unnecessary biopsies will be performed. While the alternative approach of ultrafast early perfusion imaging may potentially compensate for that, its applicability for avoiding unnecessary biopsies in a combined diagnostic model has not yet been proven.

The main limitation of this study was that the MRI scans analyzed were acquired with old protocols and on different MRI equipment, with different field strengths and sequence parameters. This was not avoidable, as patients were recruited consecutively from a longitudinal, prospective, high-risk screening study. On the other hand, this limitation can also be seen as a strength, as it corroborates the general applicability of the Kaiser score, which is based on regular BI-RADS features intended to be used independent from MRI protocols and scanning equipment. Nevertheless, the heterogeneous image quality may be the reason only a fair-to-moderate inter-reader agreement could be achieved, in contradiction to previously reported data [26, 27]. Another reason for this might be the fact that readers were not trained before the study as it was done in a previous study, further contributing to inter-reader variation [14].

In conclusion, this study provides evidence that the Kaiser score may be used in high-risk patients recalled from screening due to the detection of BI-RADS 4 lesions to avoid unnecessary biopsies, in particular those lesions presenting as masses. This has a positive potential to impact healthcare costs, as well as patient concern.

## Electronic supplementary material

Supplementary Figure 1 Patient selection flowchart (PNG 379 kb)

## Abbreviations

AUC: Area under the curve
BI-RADS: Breast imaging reporting and data system
BRCA: Breast cancer gene
CNB: Core needle biopsy
DCIS: Ductal carcinoma in situ
DWI: Diffusion-weighted magnetic resonance imaging
MIP: Maximum intensity projections
MRI: Magnetic resonance imaging
ROC: Receiver operating characteristic
STIR: Short-TI inversion recovery
VABB: Vacuum-assisted breast biopsy
VIBE: Volumetric interpolated breath-hold examination
