# Supplementary figures and images for: The Kaiser score reliably excludes malignancy in benign contrast-enhancing lesions classified as BI-RADS 4 on breast MRI high-risk screening exams
Source: Eur Radiol. 2020 Jun 6;30(11):6052–61. doi: 10.1007/s00330-020-06945-z (PMC7553895; doi:10.1007/s00330-020-06945-z)

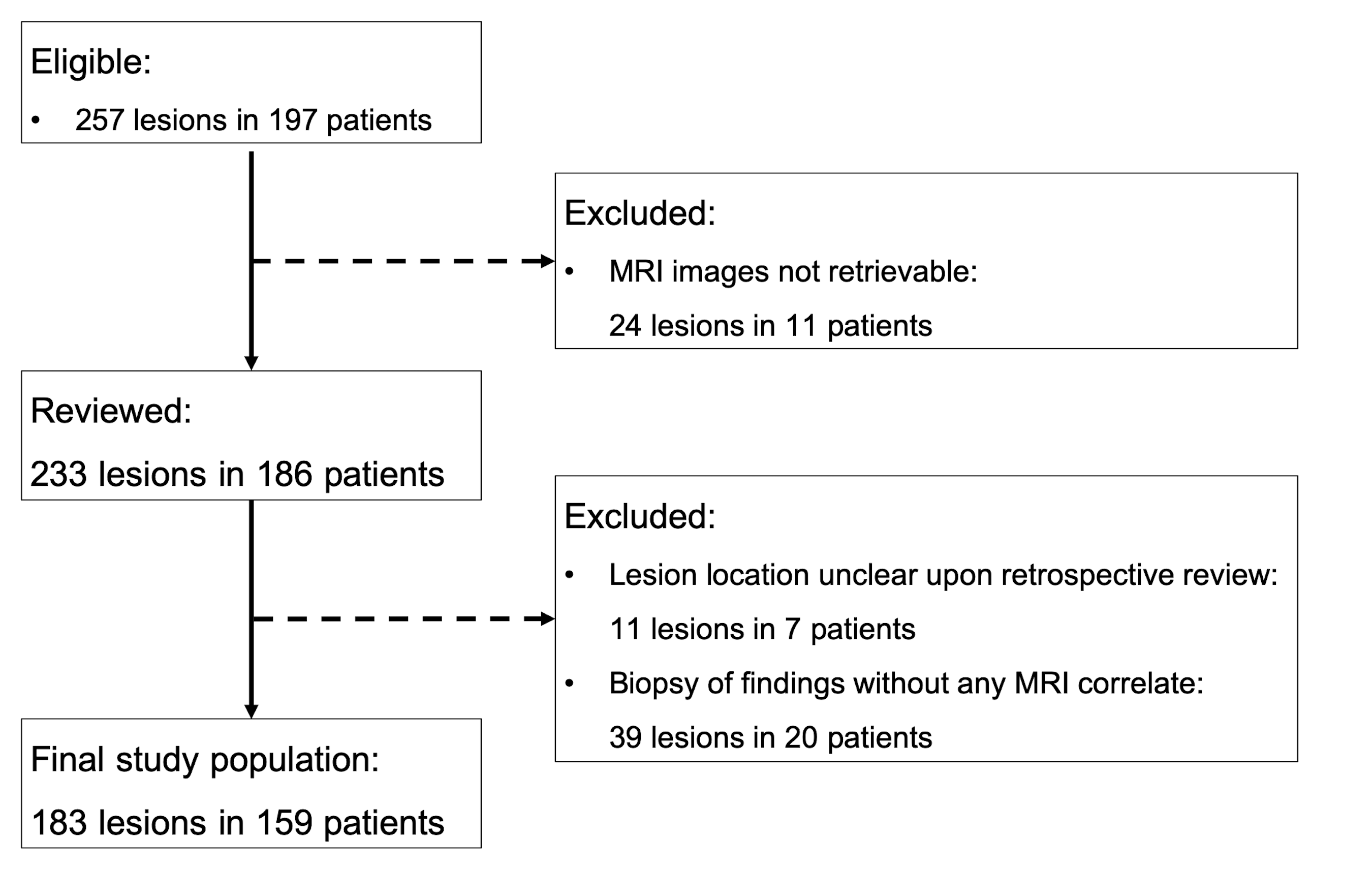

Supplement: Supplementary file 1 — Patient selection flowchart (PNG 379 kb) [file 330_2020_6945_MOESM1_ESM.png]
